# Successful percutaneous transluminal angioplasty for the treatment of renovascular hypertension with an atrophic kidney

**DOI:** 10.1007/s00380-013-0457-4

**Published:** 2014-01-03

**Authors:** Keisuke Maruyama, Junko Chinda, Maki Kabara, Naoki Nakagawa, Takayuki Fujino, Toshiharu Takeuchi, Naoyuki Hasebe

**Affiliations:** Division of Cardiology, Nephrology, Pulmonology and Neurology, Department of Internal Medicine, Asahikawa Medical University, Midorigaoka-higashi 2-1-1-1, Asahikawa, Japan

**Keywords:** Renovascular hypertension, Percutaneous transluminal renal angioplasty, Atrophic kidney, Renal vein sampling

## Abstract

Renovascular hypertension is an important cause of secondary hypertension. We present the case of a 61-year-old man with renovascular hypertension caused by chronic total occlusion of the left renal artery resulting in an atrophic kidney. Although renography indicated almost no residual function of the left kidney, renal vein sampling showed a significant increase of renin secretion in the left kidney. The endocrine function of the left kidney was believed to be preserved; thus, we performed percutaneous transluminal renal angioplasty with stent placement. After the procedure, the patient’s blood pressure decreased gradually to within the normal range without adverse events. The laboratory data on endocrine function and the renography findings drastically improved. Percutaneous transluminal renal angioplasty is a promising therapeutic procedure for renovascular hypertension with an atrophic kidney.

## Introduction

Renovascular hypertension (RVH) is an important cause of secondary hypertension. Although reports on the frequency of the disease differ, it occurs in fewer than 1 % of patients with mild to moderate hypertension [1] and in 30 % of those with acute, severe, or refractory hypertension [2]. The causes of renal artery stenosis include fibromuscular dysplasia [3], Takayasu arteritis [4], and arteriosclerosis [1, 2]. In particular, atherosclerotic renal artery stenosis is the most common cause and is increasing in frequency because of lifestyle diseases, such as hypertension, diabetes mellitus, and dyslipidemia [5]. In addition, it leads to ischemic atherosclerotic nephropathy, which accounts for 11–14 % of cases of end-stage renal disease [6]. Atherosclerotic renal artery stenosis is important not only for RVH but also as one of the manifestations of generalized atherosclerotic disease [7]. Furthermore, it is reported that the risk of cardiovascular death is increased in patients with atherosclerotic renal artery sclerosis [8].

Here we present the case of a 61-year-old man with RVH caused by chronic total occlusion of left renal artery resulting in an atrophic kidney. Although the left kidney was atrophic and seemed to be almost nonfunctional on renography, renal vein sampling showed that the endocrine function of the left kidney was preserved. Along with the findings of multidetector-row computed tomography (MDCT), we elected to perform percutaneous transluminal renal angioplasty (PTRA) with stent placement. The blood pressure decreased gradually to within the normal range after PTRA, and the laboratory data on endocrine function and the renography findings drastically improved. We present the patient’s clinical course and the effectiveness of PTRA for atrophic kidney.

## Case report

The patient was a 61-year-old man with no medical history. In October 2012, a local doctor first identified hypertension in this patient. His blood pressure was 220/110 mmHg. In addition, his laboratory data showed hypokalemia, and both the plasma renin activity (PRA) and plasma aldosterone concentration (PAC) were elevated. There was a strong suspicion of secondary hypertension, and he was therefore referred to our hospital. A few days later, he experienced general fatigue and blurry vision. He visited our hospital and was admitted as a hypertensive emergency.

**Fig. 1:**
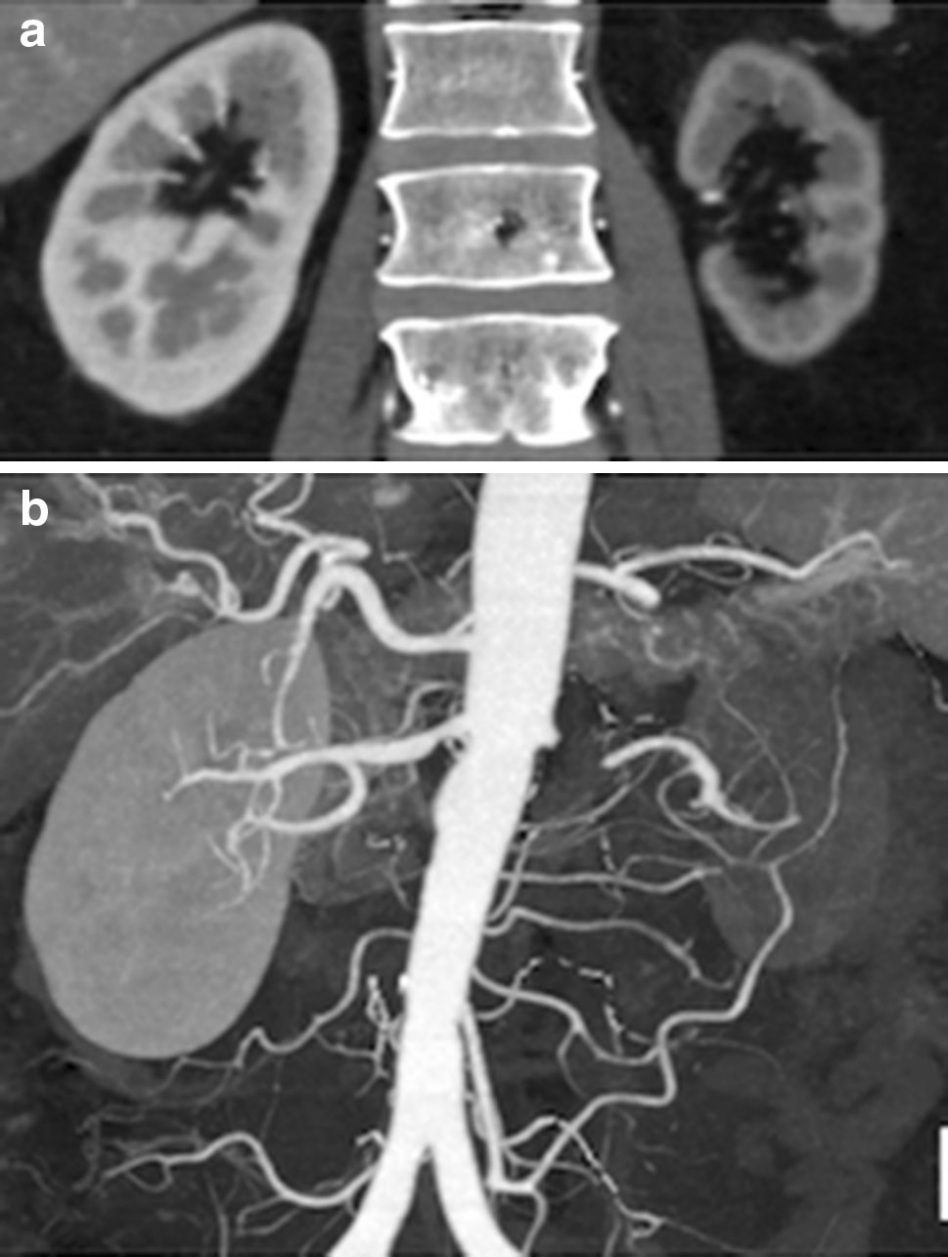
Multidetector-row computed tomography images of the kidneys and vessels demonstrating occlusion of left renal artery, atrophy of left kidney, and mild stenosis of right renal artery. **a** Coronal plane view. **b** Three-dimensional view. Note that vessels collateral to the left renal artery around the left kidney were rich

**Fig. 2:**
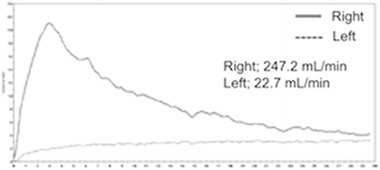
^99m^Tc-mercaptoacetyltriglycine (^99m^Tc-MAG_3_) renogram. The effective renal plasma flow of the left kidney markedly decreased and showed no peak curve

**Fig. 3:**
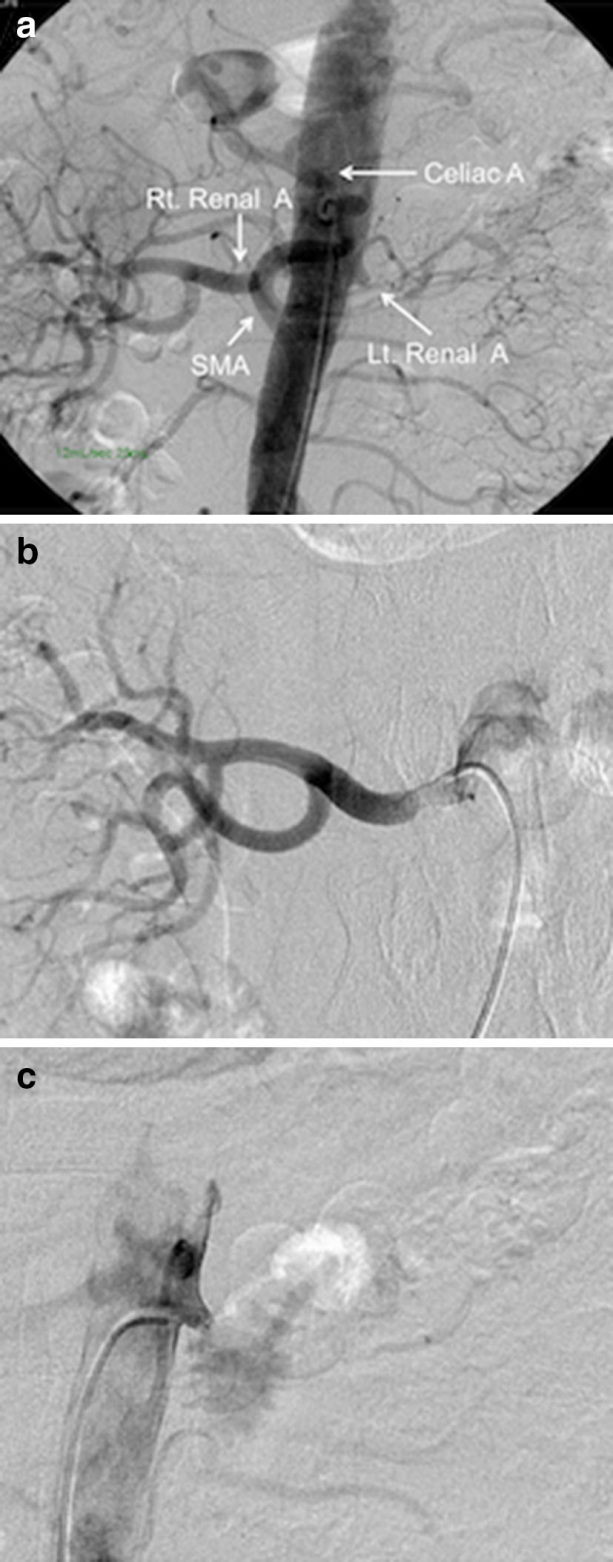
Digital subtraction abdominal and renal angiography. **a** Aortic angiography. **b** The right renal artery showing mild stenosis of the proximal portion. **c** The left renal artery showing total occlusion. *A* artery, *Rt* right, *Lt* left, *SMA* superior mesenteric artery

**Fig. 4:**
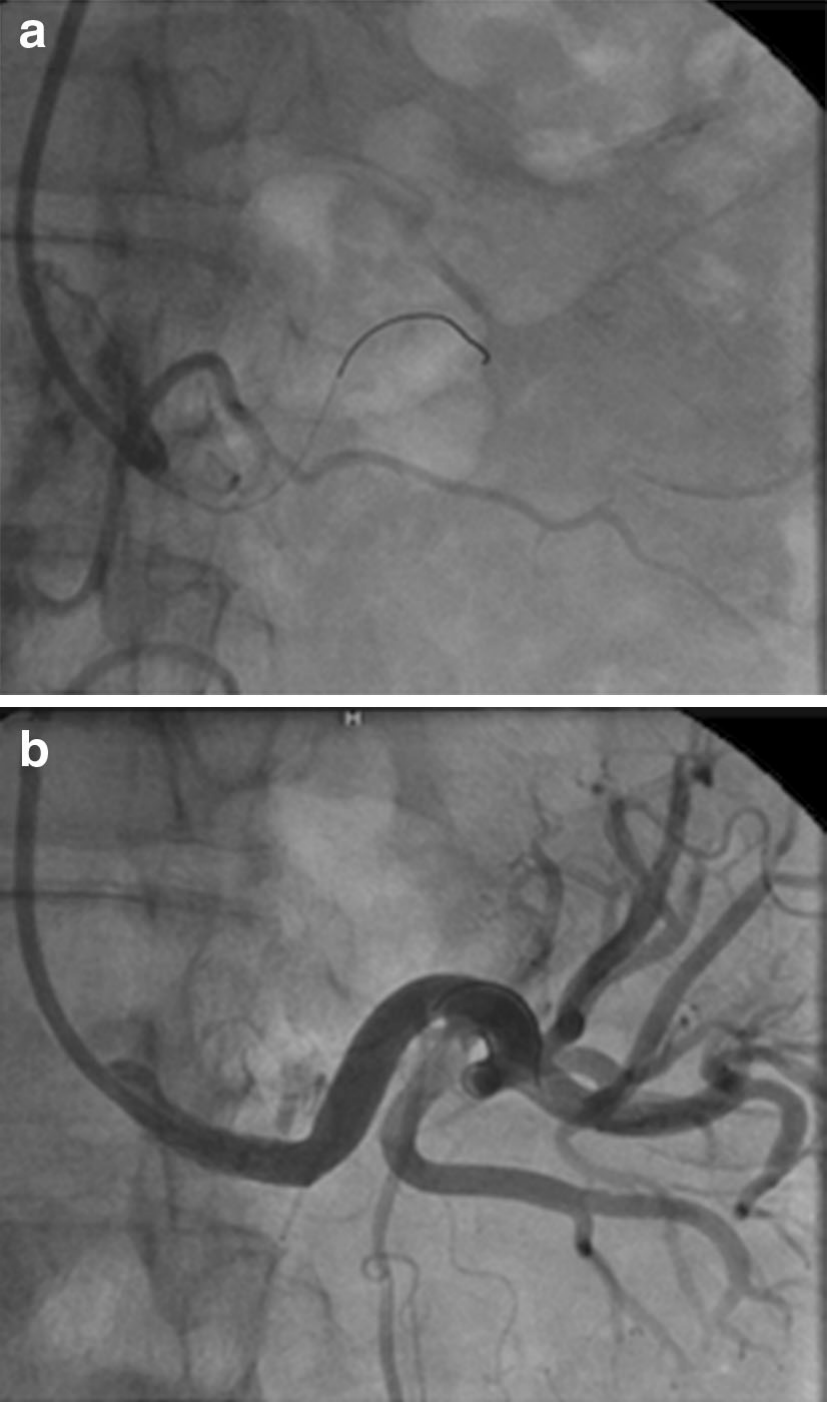
**a** Percutaneous renal angioplasty (PTRA). **b** Renal angiography after PTRA

**Fig. 5:**
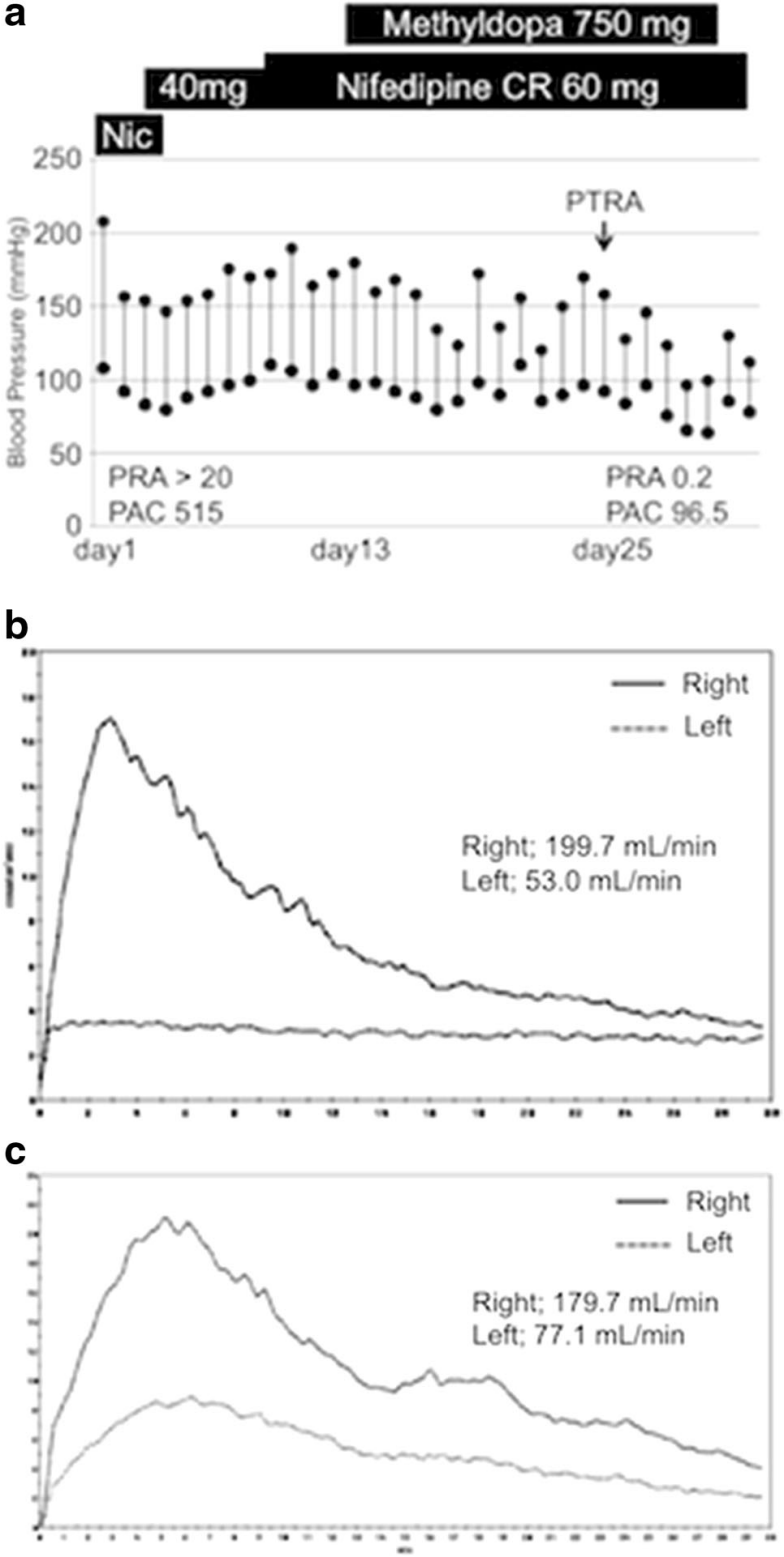
**a** Clinical course. *CR* controlled release, *PRA* plasma renin activity, *PAC* plasma aldosterone concentration, *Nic* nicardipine, *PTRA* percutaneous transluminal renal angioplasty. **b** ^99m^Tc-MAG_3_ renogram after PTRA. **c** ^99m^Tc-MAG_3_ renogram 1 year after PTRA

On admission, he had a blood pressure of 200/102 mmHg and a pulse of 78 beats/min. Physical examination revealed a systolic murmur, grade II/VI, in the left parasternal area with accentuation of the aortic second heart sound and a typical bruit in the umbilical region. The lungs were clear on auscultation, and no edema of the lower limbs was present. There were no neurologic findings except for blurry vision. On ophthalmologic examination, Keith–Wagener–Baker classification grade IV hypertensive retinopathy was noted. The laboratory data showed a blood urea nitrogen (BUN) level of 16 mg/dl, serum creatinine level of 1.04 mg/dl, and serum potassium level of 3.2 mEq/l. The C-reactive protein level was <0.10 mg/dl. PRA was >20 ng/ml/h and PAC was 515 pg/ml. Arterial blood gas analysis on room air showed a pH of 7.42, PaCO_2_ of 43 torr, PaO_2_ of 80 torr, and a bicarbonate level of 28.1 mmol/l. Based on these findings, we made a diagnosis of RVH. Chest X-ray demonstrated clear lung fields, and an electrocardiogram showed strain T waves on II, III, aV_F_, V_5_, and V_6_, as well as a high voltage (SV_1_ + RV_5_ = 50 mm). Renal ultrasonography showed that the right kidney was 12.0 × 5.7 × 5.7 cm in size and the left was 8.8 × 4.7 × 4.3 cm. We suspected stenosis of the left renal artery because of the atrophic kidney. After admission, we stabilized the blood pressure with continuous infusion of nicardipine. On the third day, the blurry vision improved. The intravenous infusion of nicardipine was changed to take controlled-release nifedipine by mouth. A dose of controlled-release nifedipine, 60 mg/day, was not successful in lowering the blood pressure, so methyldopa, 750 mg/day, was added. MDCT showed occlusion of the left renal artery, atrophy of the left kidney, and mild stenosis of the right renal artery (Fig. 1a, b). Collateral vessels to the left renal artery around the left kidney were identified, and the left renal artery was determined to have chronic total occlusion. ^99m^Tc-mercaptoacetyltriglycine (^99m^Tc-MAG_3_) renography revealed a marked decrease in the effective renal plasma flow of the left kidney (left side, 22.7 ml/min; right side, 247.2 ml/min), and no peak curve of the left kidney on renogram (Fig. 2), suggesting that the left kidney might have no residual renal function. To determine the pathophysiology, angiography and renal vein sampling were performed. Aortic angiography showed mild stenosis of the right kidney artery and total occlusion of the left kidney artery (Fig. 3a–c). The MDCT findings also suggested that collateral vessels were present. Renal vein sampling indicated that the PRA of the left vein was 3.2 times higher than that of the right vein (left, 48 ng/ml/h; right, 15 ng/ml/h). Based on these findings, we concluded that the left kidney was atrophic and had almost no residual renal function because of chronic total occlusion, but the endocrine function remained. MDCT showed that the total occlusion lesion of the left renal artery was short (length, 1.5 cm) with no calcification. Therefore, we decided to perform PTRA after informed consent to treatment and complications. A 6.5-F sheathless guiding catheter (Parent Plus PTRA; Medikit, Tokyo, Japan) was positioned at the ostial part of the left renal artery. A 0.014-inch guide wire (Shevalier; Cordis, East Bridgewater, NJ, USA) was able to cross into the distal true channel (Fig. 4a). Predilatation was performed with a 1.5-mm balloon (Make Way Plus; Kawasumi, Tokyo, Japan) at 12 atm. Further dilatation was performed with a 4-mm balloon (Aviator Plus; Cordis) at 12 atm. After predilatation, a 5.0 × 18-mm stent (Palmaz Gensis; Cordis) was deployed across the lesion at 14 atm with a good final result, and the blood flow of the left renal artery was completely recovered (Fig. 4b). There were no complications such as dissection, perforation, or embolization. The next day, blood pressure decreased gradually to 100–120/60–80 mmHg after PTRA, and methyldopa was discontinued (Fig. 5a). Before discharge, both the PRA and PAC were much lower than on admission, 0.2 ng/ml/h and 96.5 pg/ml, respectively. ^99m^Tc-MAG_3_ renography revealed a moderate decrease in the effective renal plasma flow of the right kidney and a mild increase in that of the left kidney (left side, 53.0 ml/min; right side, 199.7 ml/min) (Fig. 5b).

**Fig. 6:**
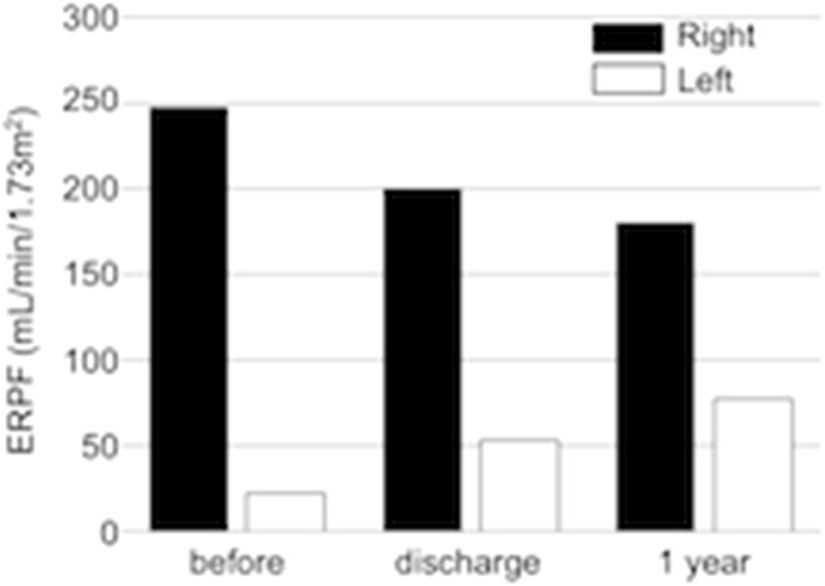
Split renal function shift after PTRA. *ERPF* effective renal plasma flow

**Fig. 7:**
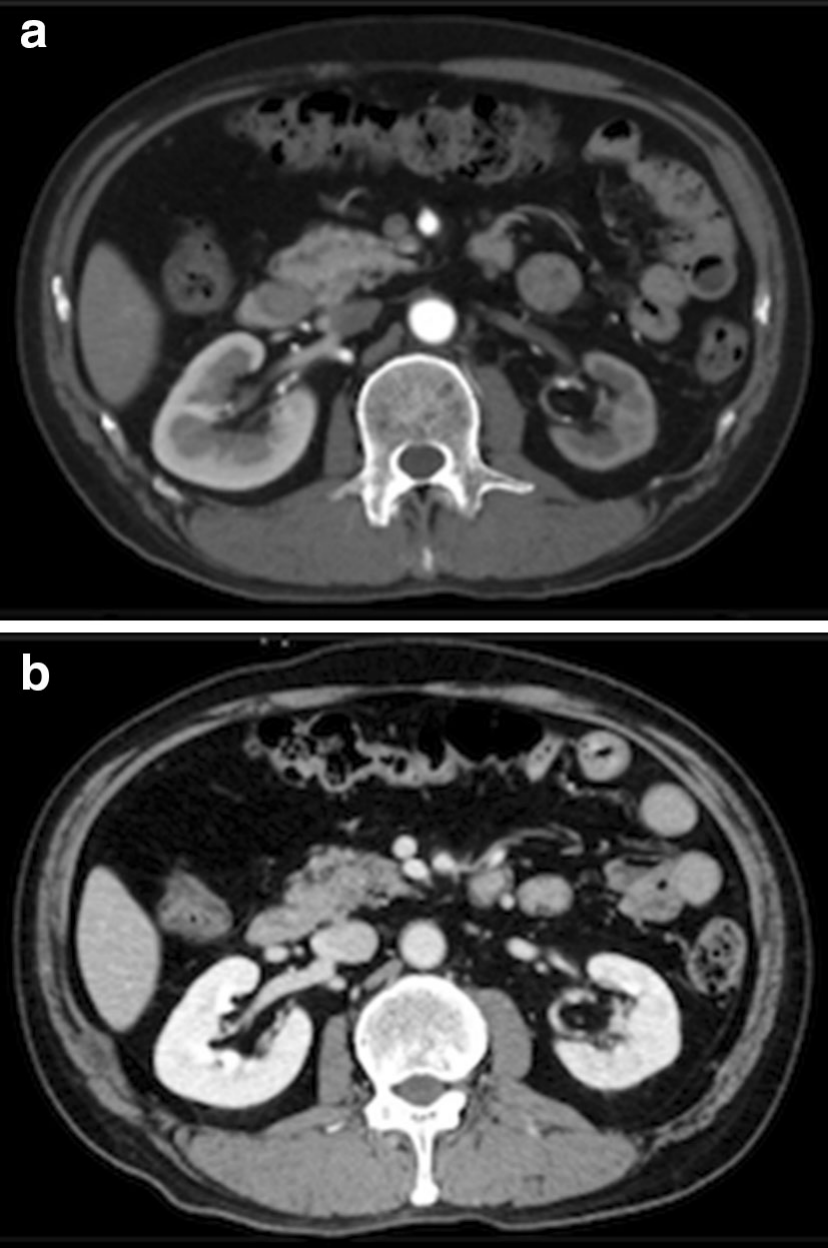
Computed tomography images of the kidneys demonstrating improvement in atrophy of the left kidney. **a** Before PTRA. **b** One year after PTRA

One year after the procedure, we re-evaluated ^99m^Tc-MAG_3_ renography and computed tomography. Compared with previous findings, the renogram revealed a peak curve of the left kidney (Fig. 5c), and further increase in the effective renal plasma flow of the left kidney (77.1 ml/min) and a decrease in that of the right kidney (179.7 ml/min) (Fig. 6). Furthermore, the computed tomogram demonstrated an improvement in the atrophy of the left kidney (Fig. 7a, b), and the kidney function also improved to a serum creatinine level of 0.74 mg/dl.

## Discussion

We report here the successful treatment of RVH caused by chronic total occlusion with an atrophic kidney, using PTRA and adjunct stent placement. The 2006 American College of Cardiology/American Heart Association guidelines state the recommendations for PTRA in patients with failure of optimal medical therapy to control blood pressure [9]. Although there was the possibility that our patient’s blood pressure could be controlled only by medication, the PTRA improved not only the blood pressure, but also both PRA and PAC. Therefore this treatment may reduce the future requirement of medications in this patient.

In patients with RVH, an accurate differential diagnosis is important. First, we excluded Takayasu arteritis because there was no sign of fever and a C-reactive protein level within normal limits. Next, we considered that the possibility of fibromuscular dysplasia was low because the lesion was in the proximal portion of the renal artery, which is not common in patients with fibromuscular dysplasia. In addition, the patient lacked other atherosclerotic lesions, i.e., carotid arteries [3]. Third, we considered the possibility of an acute kidney infarction, but MDCT findings did not suggest a partial perfusion defect and there was no arrhythmia such as atrial fibrillation on ambulatory electrocardiogram. Moreover, there was no flank or abdominal pain, or other symptoms including nausea and vomiting [10]. Therefore, based on the MDCT findings that the collateral vessels were rich, we reached the diagnosis of RVH caused by arteriosclerosis.

Although the therapeutic approach in RVH remains controversial, there are three therapeutic options available: medical therapy, percutaneous angioplasty with or without stent placement, and surgical revascularization or, in some cases, resection of a “pressor” kidney. The effectiveness of angiotensin-converting enzyme and angiotensin II receptor blockers has made it easy to control blood pressure in patients with RVH [11]. On the other hand, several studies showed the effectiveness of PTRA with or without stent placement [12, 13]. A prospective study investigating the efficacy of stent revascularization for the prevention of cardiovascular and renal events among patients with renal artery stenosis is ongoing [14].

The management of RVH in patients with atrophic kidney is unclear. Thomaz et al. [15] demonstrated the beneficial effect on blood pressure of nephrectomy of the atrophic kidney. However, nephrectomy is normally accepted as one of the last options. Nagata et al. [16] first reported successful PTRA with stent placement in a patient with a chronic total occluded renal artery in an atrophied kidney. In this case, renal vein sampling was carried out and showed bilateral difference; this was similarly observed in our case. Alchi et al. [17] also evaluated the bilateral difference in a case of chronic renal artery occlusion. These two cases and our case indicate the possibility that the renin–angiotensin system is enhanced in an atrophic kidney with chronic renal artery occlusion. Therefore, PTRA with stent placement might be useful in a case with residual endocrine function that is maintained by collateral blood flow in an atrophic kidney with chronic renal artery occlusion.

In the present case, the effective renal plasma flow increased in the left kidney with chronic total occlusion and decreased in the right kidney with mild stenosis, as determined by ^99m^Tc-MAG_3_ renography after PTRA. This result was even more noticeable 1 year after PTRA. La Batide-Alanore et al. [18] reported similar outcomes after PTRA in 32 patients with unilateral renal artery stenosis. It is unclear whether the treatment is associated with long-term renal benefits; however, the decrease in effective renal flow in the intact kidney leads to a reduction of the intraglomerular pressure, which surely results in protection of the kidney. In fact, the kidney function in this patient improved to within the normal range.

Most interestingly, computed tomography demonstrated improvement in the atrophy of the left kidney. Whereas the atrophy in itself is considered to be irreversible in general, it was reversible in the present case. Mounier-Véhier et al. [19] have also reported that there was a significant increase in medullary length in the poststenotic/revascularized kidneys at 6 months after PTRA in renovascular hypertensive patients, suggesting that the increase in the effective renal plasma flow may be related to the improvement in the size of atrophic kidney. These findings could provide useful knowledge in considering the indications for PTRA.

In conclusion, we presented a case of chronic total occlusion in an atrophic kidney treated with PTRA. Further studies are required to confirm the efficacy of PTRA for RVH with an atrophic kidney.
